# Clinical, biochemical and metabolic characterization of patients with short-chain enoyl-CoA hydratase(ECHS1) deficiency: two case reports and the review of the literature

**DOI:** 10.1186/s12887-020-1947-z

**Published:** 2020-02-03

**Authors:** Hua Yang, Dan Yu

**Affiliations:** 1 Department of Pediatrics, West China Second University Hospital, Ren Min South Road 3rd Second 20#, Chengdu, 610041 Sichuan China; 2 Key Laboratory of Obstetric & Gynecologic, Pediatric Diseases and Birth Defects of Ministry of Education, Chengdu, China

**Keywords:** Short-chain enoyl-CoA hydratase deficiency, ECHS1,urinary metabolic profile

## Abstract

**Background:**

Short-chain enoyl-CoA hydratase (SCEH or ECHS1) deficiency is a rare congenital metabolic disorder caused by biallelic mutations in the ECHS gene. Clinical phenotype includes severe developmental delay, regression, dystonia, seizures, elevated lactate, and brain MRI abnormalities consistent with Leigh syndrome (LS). SCEH is most notably involved in valine catabolism. There is no effective treatment for the disease, patients may respond to dietary restriction of valine and supplementation of N-acetylcysteine .

**Case presentation:**

We describe two patients who presented in infancy or early childhood with SCEH deficiency. Both patients were shown to harbor heterozygous or homozygous variants in the ECHS1 gene, and developmental retardation or regression as the onset manifestation. Brain MRI showed abnormal signals of bilateral pallidus. Urine metabolic examination showed increased levels of 2,3-dihydroxy-2-methylbutyric acid and S-(2-carboxypropyl) cysteamine S-(2-carboxypropoxypropyl) cysteamine (SCPCM). A valine restricted diet and combined of N-acetylcysteine supplementation were utilized in the two patients.

**Conclusions:**

In clinical practice, The elevated urinary 2,3-dihydroxy-2-methylbutyrate, S-(2-carboxypropyl) cysteine, S-(2-carboxypropyl) cysteine and N-acetyl-S-(2-carboxypropyl) cysteine levels might be clues for diagnosis of SCEH deficiency which can be confirmed throughGenetic sequencing of ECHS1 gene. Early cocktail therapy, valine restrictied diet and N-acetylcysteine supplementation could improve the prognosis of patients.

## Background

Short-chain enoyl-CoA hydratase(SCEH or ECHS1) deficiency, also known as crotonase deficiency, is a rare autosomal recessive hereditary disease (OMIM 616277). It was first reported by Peters et al. (2014)and caused by pathogenic variants in the ECHS1 gene [1]. Clinical manifestations include mental retardation or degeneration, dystonia, seizure, hyperlacticemia, elevated lactate, and brain MRI abnormalities consistent with Leigh syndrome (LS). Theincidence of SCEH deficiency is very low, and there are only 44 cases reported abroad (Table 1) and at present, no SCEH deficiency has been reported in China. In this study, the clinical data of two patients with SCEH deficiency were reported, and the relevant literatures were reviewed to improve clinicians’ understanding of this disease.

**Table 1:** Clinical features of previously reported patients with ECHS1 deficiency

Reference	This report		Peters et al.201 4[1]		Sakai et al.2014 [2]	Haack et al.2015 [3]				
Patient ID	Patient 1	Patient 2	Patient 1	Patient 2	Paient 1	F1,II:2	F2,II:1	F3,II:6	F4,II:1	
Genetic mutation Protein effect	c.161G>A/c.414 + 1G>A p.Arg54His/−	c.74G>A/c.74G>A p.R25H/p.R25H	c.473C > A/c.414 + 3G > C p.Ala158Asp/splicing	c.473C > A/c.414 + 3G > C; p.Ala158Asp/splicing	c.2 T > G’/c.5C > T; p.Met1Arg/p.Ala2Val	c.176A > G/c.476A > G; p.Asn59Ser/p.Gln159Arg	c.197 T > C/c.449A > G; p.Ile66Thr/p.Asp150Gly	c.476A > G/c.476A > G; p.Gln159Arg/p.Gln159Arg	c.161G > A/c.817A > G; p.Arg54His/p.Lys273Glu	
Gender	F	M	F	M	M	F	M	F	M	
Age of onset;	8 months	2 years	Birth	Birth	2 months	Birth	Birth	Birth	Birth	
Death	Alive at 2 years	Alive at 5.2 years	4 months	8 months	Alive at 4 years	4 months	11 months	2.3 years	7.5 years	
Developmentaldelay/regression	+	+	NL	+	+	NL	+	+	+	
Hearing loss	–	–	NL	NL	+	+	+	NL	NL	
Optic atrophy	ND	–	NL	NL	NL	NL	+	NL	NL	
Epilepsy	–	–	NL	NL	NL	+	+	+	+	
Dystonia	+	+	NL	NL	NL	NL	+	+	+	
Nystagmus	+	–	NL	+	+	NL	NL	NL	NL	
Cardiomyopathy	ND	ND	–	HCM	–	HCM	HCM	ND	–	
MRI: basal ganglia T2 hyperintensity	+	+	+	–	+	+	+	+	NL	
MRS: lactate	+	ND	+	+	NL	NL	+	–	–	
Elevated plasmalactate	+	–	+	+	+	+	+	+	NL	
Elevated pyruvate	+	–	+	+	NL	NL	NL	ND	ND	
Urinary 2-methyl, 2,3-dihydroxybutyrate	+	+	+	+	NL	ND	–	ND	ND	
SCPCM/SCPC/ N-acetyl-SCPC	ND	SCPCM+	SCPCM+;SCPC+	SCPCM+;SCPC+	ND	ND	ND	ND	ND	
Reference	Haack et al.2015 [3]						Ferdinandusse et al.2015 [4]			
Patient ID	F5,II:3	F6,II:1	F7,II:2	F8,II:1	F9,II:2	F10,II:1	Patient 1	Patient 2	Patient3	
Genetic mutation Protein effect	c.673 T > C/c.673 T > C; p.Cys225Arg/p.Cys225Arg	c.98 T > C/c.176A > G; p.Phe33Ser/p.Asn59ser	c.268G > A/c.583G > A; p.Gly90Arg/p.Gly195Ser	c.161G > A/c.394G > A; p.Arg54His/p.Ala132Thr	c.161G > A/c.431up; p.Arg54His/p.Leu145Alafs*6	c.229G > C/c.476A > G; p.Glu77Gln/p.Gln159Arg	c.817A > G/c.817A > G; p.Lys273Glu/p.Lys273Glu	c.817 > G/c.817A > G; p.Lys273Glu/p.Lys273Glu	c.433C > T/c.476A > G; p.Leu145Phe/p.Gln159Arg	
Gender	F	F	F	F	F	F	F	F	F	
Age of onset;	Birth	Birth	2 years	1 Year	Birth	11 months	Birth	Birth	Early infancy	
Death	Alive at2 years	Aliveat 3 years	Alive at 5 years	Alive at 8 years	Alive at 16 years	Alive at 31 years	24 h	2 days	Alive at 7 years	
Developmentaldelay/regression	+	+	+	+	+	+	NL	NL	+	
Hearing loss	NL	+	+	+	+	+	NL	NL	+	
Optic atrophy	NL	NL	–	–	+	+	NL	NL	+	
Epilepsy	+	+	–	+	–	+	NL	NL	NL	
Dystonia	–	+	+	–	+	+	NL	NL	+	
Nystagmus	NL	NL	NL	NL	+	+	NL	NL	NL	
Cardiomyopathy	HCM	DCM	ND	NL	–	–	+	–	NL	
MRI: basal ganglia T2 hyperintensity	+	+	+	NL	+	+	NL	+	NL	
MRS: lactate	–	NL	+	NL	+	–	NL	NL	NL	
Elevated plasmalactate	+	+	–	+	+	+	+	+	+	
Elevated pyruvate	+	ND	–	NL	NL	NL	+	+	ND	
Urinary 2-methyl, 2,3-dihydroxybutyrate	+	ND	+	ND	ND	–	+	+	+	
SCPCM/SCPC/ N-acetyl-SCPC	ND	ND	ND	SCPCM+	SCPCM+	ND	ND	ND	SCPCM+;SPCP+	
Reference	Ferdinandusse et al.2015 [4]	Tetreault et al.2015 [5]				Yamada et al.2015 [6]		Ganetzky et al.2016 [7]		
Patient ID	Patient4	P1	P2	P3	P4	III-2	III-3	Patient 1	Patient 2	
Genetic mutation Protein effect	c.673 T > C/c.674G > C; p.Cys225Arg/p.Cys225Ser	c.538A > G/c.583G > A; p.Thr180Ala/p.Gly195Ser	c.538A > G/c.713C > T; p.Thr180Ala/p.Ala238Val	c.538A > G/c.713C > T; p.Thr180Ala/p.Ala238Val	c.538A > G/c.476A > G; p.Thr180Ala/p.Gln159Arg	c.176A > G/c.413C > T; p.Asn59Ser/p.Ala138Val	c.176A > G/c.413C > T; p.Asn59Ser/p.Ala138Val	c.8C > A/c.389 T > A; p.Ala3Asp/p.Val130Asp	c.8C > A/c.389 T > A; p.Ala3Asp/p.Val130Asp	
Gender	M	F	M	M	F	F	M	M	F	
Age of onset;	1 year	2.5 months	2.9 years	10 months	6 months	10 months	7 months	Prenatal	Prenatal	
Death	Alive at 1 year	10 months	Alive at 18 years	Alive at 12 years	Alive at 12 years	Alive at 7 years	5 years	16 h	24 h	
Developmental delay/regression	+	+	+	+	+	+	+	NL	NL	
Hearing loss	NL	–	+	+	+	–	NL	NL	NL	
Optic atrophy	+	–	+	+	+	NL	NL	NL	NL	
Epilepsy		NL	NL	NL	NL	–	–	NL	NL	
Dystonia	+	–	+	–	+	+	+	NL	NL	
Nystagmus	NL	+	+	+	+	NL	NL	NL	NL	
Cardiomyopathy	NL	NL	NL	NL	NL	ND	ND	DCM	DCM	
MRI: basal ganglia T2 hyperintensity	+	+	+	+	+	+	+	NL	NL	
MRS: lactate	+	+	–	–	–	NL	NL	NL	NL	
Elevated plasmalactate	+	+	+	+	–	–	–	+	+	
Elevated pyruvate	ND	NL	NL	NL	N/A	+	NL	+	+	
Urinary 2-methyl, 2,3-dihydroxybutyrate	+	ND	ND	ND	ND	–	+	ND	+	
SCPCM/SCPC/ N-acetyl-SCPC	SCPCM+;SPCP+	ND	ND	ND	ND	N-acetyl-SPCPM+	N-acetyl-SPCPM+	ND	ND	
Reference	Nair et al.2016 [8]	Olgiati et al.2016 [9]		Mahajan et al.2017 [10]	Bedoyan etal.2017 [11]	Huffnagel etal.2017 [12]	Al Mutairi et al.2017 [13]		Balasubramaniam et al.2017 [14]	
Patient ID	Patient 1	II-1	II-2	Patient1	Patient1	Patient1	Patient 1	Patient 2	Patient1	
Genetic mutation Protein effect	c.842A > G/c.842A > G; p.Glu281Gly/p.Glu281Gly	c.232G > T/c.518C > T; p.Glu78Ter/p.Ala173Val	c.232G > T/c.518C > T; p.Glu78Ter/p.Ala173Val	c.518C > T/c.817A > G; p.Ala173Val/p.Lys273Glu	c.836 T > C/c.8C > A; p.Phe279Ser/p.Ala3Asp	c.229G > C/c.563C > T p.Glu77Gln/p.Ala188Val	c.88 + 5G > A/c.88 + 5G > A; p.Ala31Glufs*23/p.Ala31Glufs*23	c.88 + 5G > A/c.88 + 5G > A; p.Ala31Glufs*23/p.Ala31Glufs*23	c.476A > G/c.538A > G; p.Gln159Arg/p.Thr180Ala	
Gender	F	M	M	M	M	F	F	M	F	
Age of onset;	Birth	3.5 years	4.5 years	8 years	Birth	6 weeks	Birth	Birth	17 months	
Death	24 h	Alive at 17 years	Alive at 15 years	Alive at 8 years	39 days	Alive at 26 years	2 days	8 h	Alive at 4.5 years	
Developmentaldelay/regression	+	+	–	NL	NL	+	NL	NL	+	
Hearing loss	NL	NL	–	NL	+	–	NL	NL	–	
Optic atrophy	NL	NL	–	NL	–	+	NL	NL	–	
Epilepsy	NL	–	–	NL	NL	–	NL	NL	NL	
Dystonia	NL	+	+	+	+	+	NL	NL	+	
Nystagmus	NL	NL	–	NL	NL	+	NL	NL	NL	
Cardiomyopathy	–	NL	NL	NL	ND	–	ND	ND	NL	
MRI: basal ganglia T2 hyperintensity	NL	+	+	+	+	+	ND	ND	+	
MRS: lactate	NL	–	NL	NL	+	–	ND	ND	NL	
Elevated plasmalactate	+	–	–	–	+	+	+	+	+	
Elevated pyruvate	–	–	–	ND	+	–	+	NL	–	
Urinary 2-methyl, 2,3-dihydroxybutyrate	+	–	–	ND	+	ND	ND	ND	ND	
SCPCM/SCPC/ N-acetyl-SCPC	ND	SPCPM+; N-acetyl-SPCPM+	SPCPM+; N-acetyl-SPCPM+	ND	ND	SCPCM+;SCPC+; N-acetyl-SCPC+	ND	ND	ND	
Reference	Ogawa et al.2017 [15]				Fitzsimons et al.2018 [16]				Carlston et al.2018 [17]	Shayota et al.2018 [18]
Patient ID	Pt376	Pt536	Pt1038	Pt1135	Patient 1	Patient 2	Patient 3	Patient 4	Patient 1	Patient1
Genetic mutation Protein effect	c.98 T > C/c.176A > G; p.Phe33Ser/p.Asn59Ser	c.5C > T/c.1A > G; p.Ala2Val/p.Met1Val	c.5C > T/c.176A > G; p.Ala2Val/p.Asn59Ser	c.5C > T/c.176A > G; p.Ala2Val/p.Asn59Ser	c.476A > G,/c.476A > G; p.Gln159Arg/p.Gln159Arg	c.538A > G/ c.538A > G, p.Thr180Ala/p.Thr180Ala	c.538A > G/ c.538A > G, p.Thr180Ala/p.Thr180Ala	c.538A > G/ .538A > G, p.Thr180Ala/p.Thr180Ala	c.79 T > G/c.789_790del; p.Phe27Val/p.Phe263fs	c.538A > G/c.444G > T p.T180A/p.M148I
Gender	NL	NL	NL	NL	M	M	F	M	M	M
Age of onset;	NL	NL	NL	NL	5 months	3 months	5 months	2 weeks	1 year	2.5 months
Death	NL	NL	NL	NL	4 years	21 months	28 months	13 months	9 years	Alive at 15 months
Developmentaldelay/regression	NL	NL	NL	NL	+	+	+	+	+	+
Hearing loss	NL	NL	NL	NL	NL	NL	NL	NL	+	ND
Optic atrophy	NL	NL	NL	NL	NL	NL	NL	NL	–	ND
Epilepsy	NL	NL	NL	NL	+	+	+	+	–	+
Dystonia	NL	NL	NL	NL	+	+	+	+	–	+
Nystagmus	NL	NL	NL	NL	+	NL	NL	NL	NL	NL
Cardiomyopathy	ND	ND	ND	ND	ND	ND	ND	ND	ND	ND
MRI: basal ganglia T2 hyperintensity	ND	ND	ND	ND	+	+	+	+	+	+
MRS: lactate	ND	ND	ND	ND	+	+	–	+	+	NL
Elevated plasmalactate	ND	ND	ND	ND	+	+	+	+	–	+
Elevated pyruvate	ND	ND	ND	ND	ND	ND	ND	ND	–	
Urinary 2-methyl, 2,3-dihydroxybutyrate	ND	ND	ND	ND	+	+	+	+	–	ND
SCPCM/SCPC/ N-acetyl-SCPC	ND	ND	ND	ND	SPCP+	ND	ND	SPSP+	ND	ND

*NL,* not listed;*ND,* not determined, *HCM* hypertrophic cardiomyopathy, *DCM* dilated cardiomyopathy, *SCPCM* S-(2-caboxypropyl)cysteamine, *SCPC* S-(2-carboxypropyl)cysteine, *N-acetyl-SCPC* N-acetyl-S-(2-carboxypropyl)-cysteine (N-acetyl-methacryl-l-cysteine

## Case presentation

### Case 1

**Fig. 1:**
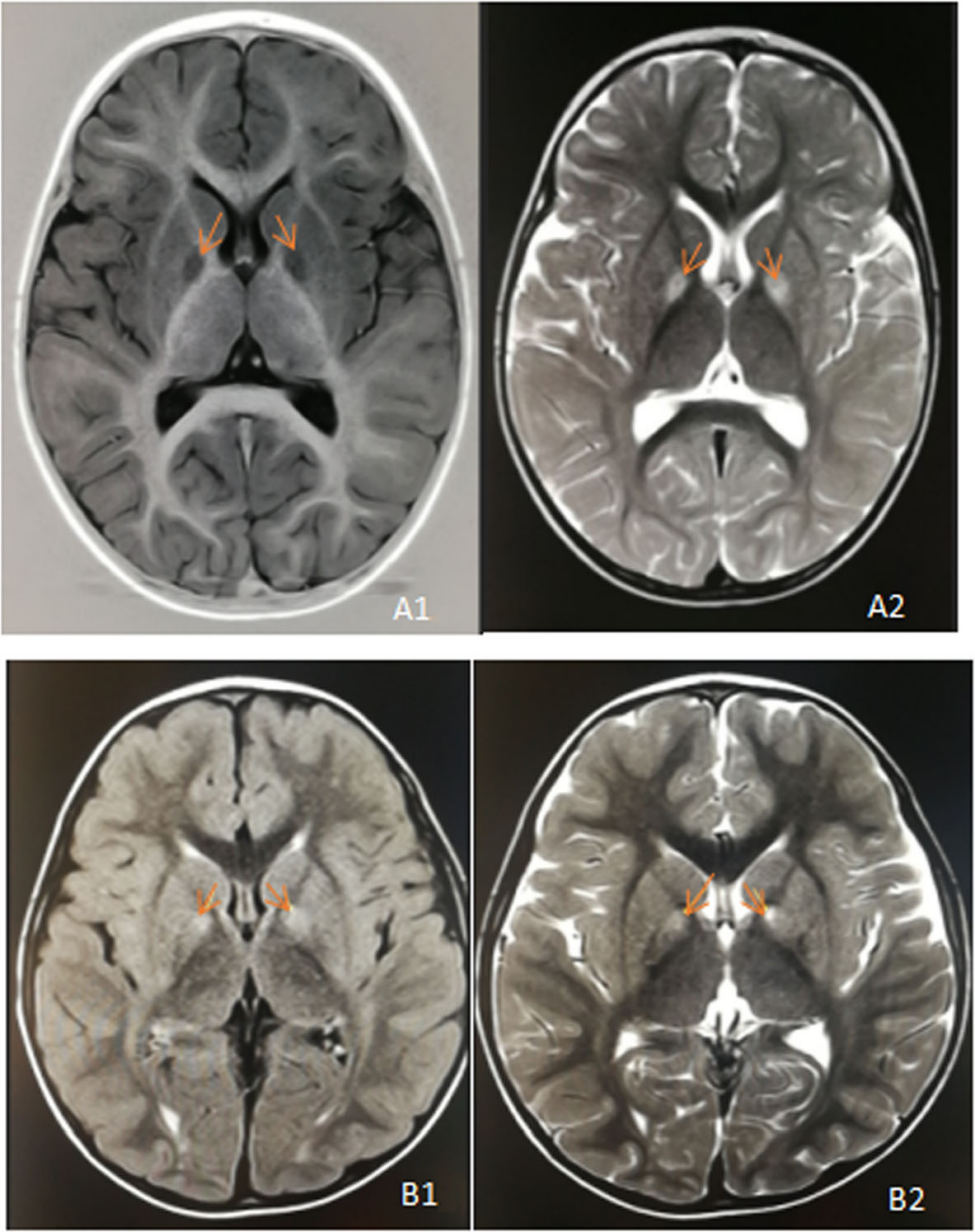
**a** Brain MRI of patient 1 showed bilateral globus pallidus swelling and abnormal signal (as shown by arrow), T1 W1 (A1) showed low signal and T2 W1 (A2) showed high signal; **b** Brain MRI of patient 2 showed a little patchy abnormal signal of bilateral globus pallidus (as shown by arrow), small patchy shadow of left globus pallidus adjacent to anterior limb of internal capsule showed slightly obvious, and both T2WI-flair(B1) and T2WI(B2)showed high signal

The child was a 2 year- old girl. She was born by caesarian section for fetal transverse presentation at term with birth weight 3.25 kg (percentile 50th–75th), height 48 cm (percentile 10th–25th), and head circumference 34 cm (percentile 50th). Her birth history was unremarkable and her early milestones were appropriate. From 8 months of age onwards motor developmental delay was noted. By 1 year of age, she had obvious developmental regression due to infection, but symptoms improved slightly after the infection was treated. The patient was first evaluated in our hospital at 14 months due to severe developmental delay. She was unable to sit alone or crawl, accompanied by language dysfunction, poor fine motion, and taking simple instructions. At this time, the patient growth parameters were: weight of 10 kg (percentile 3th–10th), length of 83 cm (percentile 10th–25th), and head circumference of 46.5 cm (percentile 25th–50th). Developmentally, she had hypotonia, language development delay, mental retardation and nystagmus. Elevated levels of blood lactate 4.84 mmol/L (normal 0.7–2.1 mmol/L), pyruvic acid122μmol/L (normal 20–100 mmol/L), and TSH3UL1.081 mTU/L(normal 1.7–9.1 mTU/L)were noted. Blood ammonia, blood routine, β-hydroxybutyric acid, ceruloplasmin, blood calcium/phosphorus/alkaline phosphatase, serum vitamin D, liver and kidney function, electrolytes were all normal. Urine organic acid analysis and electroencephalogram (EEG) in 2018 was normal, brain MRI (Fig. 1a, A2) showed bilateral pale globular morphology and signal changes which suggested the possibility of genetic metabolic diseases. MRS showed bilateral lesions with inverted lactate peaks. Re-examination of urine metabolism in 2019 showed that 2,3-dihydroxy-2-methylbutyric acid was detected significantly.

**Fig. 2:**
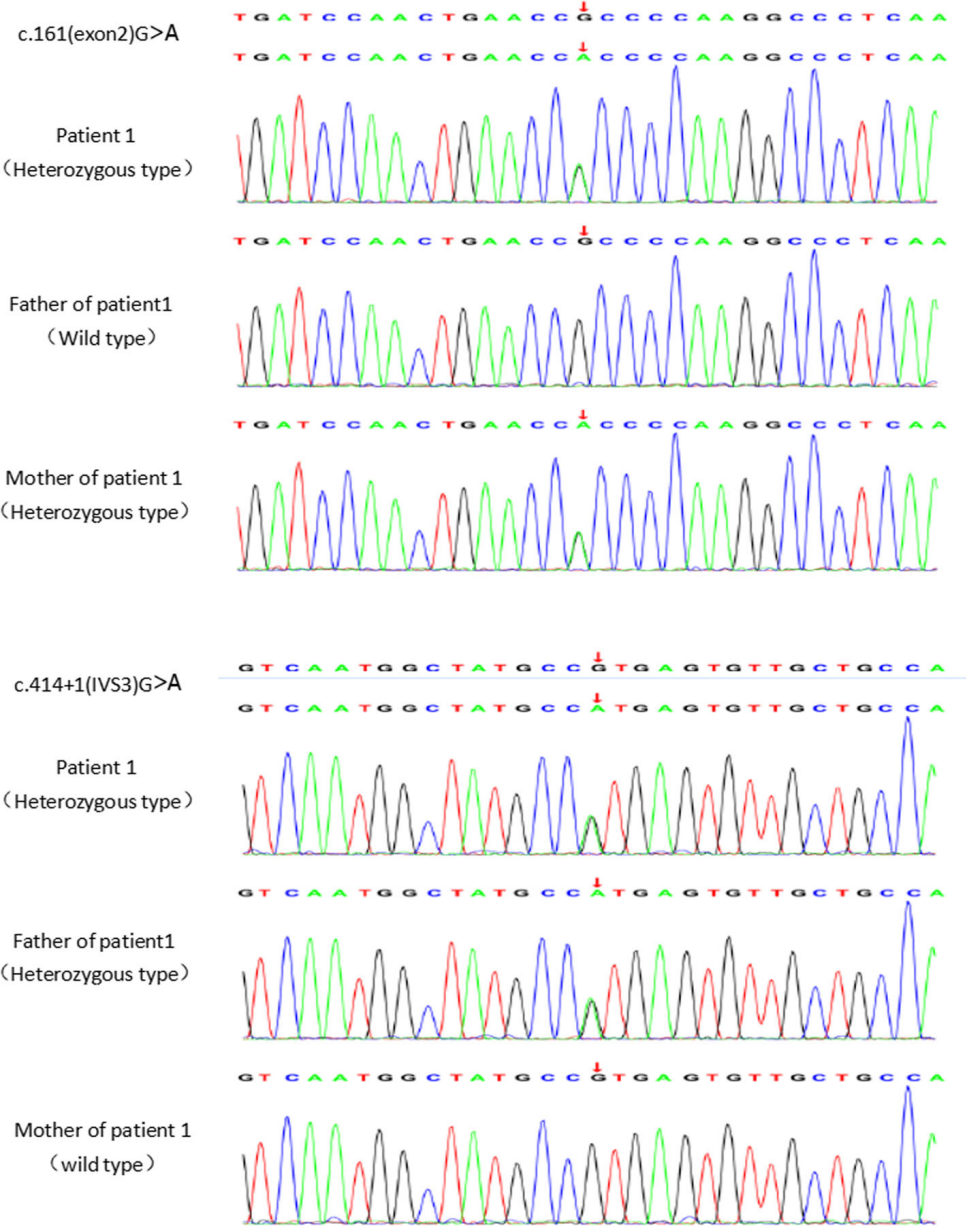
Gene detection of patients 1 and their parents in total exome gene detection. The ECHS1 gene of patient 1 had c.161G > A and c.414 + 1G > A compound heterozygous mutation, the ECHS1 gene of his mother had c.161G > A heterozygous mutation, and the ECHS1 gene of his father had c.414 + 1G > A heterozygous mutation

The genomic DNA was isolated from blood and processed for IDT The xGen Exome Research Panel on Hiseq-Illumina NGS platform. Whole exome sequencing analysis (Fig. 2) revealed that there were ECHS1 gene heterozygous mutations following: c.161G > A in exon 2 in the chr10:135184189 region and c.414 + 1G > A exists in intron 3 in the chr10:135183407 region, which were inherited from her mother and father respectively. The c.161G > A (p.Arg54His) has been previously reported in compound heterozygous state in clinically affected patients (Haack et al., 2015), and which may be pathogenic. c.414 + 1G > A genes were not reported in ClinVar, OMIM and HGMD databases, and Mutation Taster suggested the variant to be disease-causing.

### Case 2

Patient number 2 was a 5 year- old boy. He was born at 39 weeks of gestation by normal vaginal delivery after an uneventful pregnancy and the product of the second pregnancy of his mother (spontaneous abortion of the first fetus). His birth weight was 3.3 kg (percentile 25th–50th), height 49 cm (percentile 10th–25th), head circumference33.5 cm (percentile 25th–50th) with normal Apgar scores. There were no metabolic disturbances observed post-delivery. Developmental milestones were normally reached. Unsteady gait was noticed at 2 years of age with developmental regression, foot-pad walking, unable to run and jump, involuntary movement, poor fine activity, hypermyotonia and language dysfunction. His growth parameters were a weight of 11.5 kg (percentile 10th–25th), length of 85 cm (percentile 10th–25th), and head circumference of 48 cm (percentile 25th–50th). Elevated levels of α-hydroxybutyrate dehydrogenase 223 U/L (normal 2–25 U/L), lactate dehydrogenase 266 U/L (normal 106–211 U/L), creatine kinase isoenzyme 36 U/L (normal 0–25 U/L) were noted. Blood lactic acid, blood ammonia, pyruvic acid, blood routine, blood gas analysis, liver and kidney function, electrolytes were all normal. No abnormalities were found in the serum tandem mass spectrometry and urinary organic acid analysis in2017. Brian MRI (Fig. 1B1, B2) showed a few abnormal signals in the bilateral globus pallidus, and slightly obvious shadows in the left globus pallidus. Meanwhile, EEG was normal. Re-screening of urinary metabolism in 2019 suggested that 2, 3-dihydroxy-2-methylbutyric acid was slightly increased. After conducting further special metabolic examination of the urine, it was found that the concentration of S-(2-caboxypropyl) cysteamine (SCPCM) was 2.83 micromol/mmolCr, which is about four times the upper limit of reference value.

Detection of whole exome sequencing of genomic DNA showed that there was a c.74G > A mutation in the chr10:135186764 region (exon 1) of ECHS1 gene. Family sequencing data showed that his mother carried the above-mentioned heterozygous mutation. High-throughput sequencing data indicated that there was a deletion of heterozygosity in his father. c.74G > A was not reported in ClinVar, OMIM and HGMD, ExAC, dpSNP and other databases, and the missense mutation may be pathogenic.

## Discussion and conclusion

SCEH(also known as crotonase) is a 290 amino acid protein encoded by ECHS1 on chromosome 10 and localized in the mitochondrial matrix as a 160 kD ahomohexameric enzyme [4, 19]. SCEH has broad substrate specificity for acyl-CoAs with chain lengths up to 10 carbon atoms, although it shows greatest activity towards crotonyl-CoA. In the metabolic pathway, the functions of SCEH is to hydrate the double bond between the second and third carbon of enoyl-CoAs. In addition, its function in the oxidation of short chain acyl-CoAs, SCEH also has activity on the degradation intermediates of branched-chain acyl-CoAs, particularly valine, isoleucine and leucine. However, recent functional studies have shown that SCEH is a crucial enzyme in valine metabolism, but not isoleucine metabolism, and has limited effect on mitochondrial fatty acid oxidation [11, 13].

Pathogenic variants of biallelic allele in ECHS1 leads to the decrease of SCEH activity,and results in clinical manifestations of Leigh syndrome with early onset of hypotension, growth retardation or degeneration, cardiomyopathy, dystonia, sensorineural hearing loss, hypotension and malnutrition. Blood and cerebrospinal fluid (CSF) lactate and pyruvate levels are usually elevated. Brain MRI may show white matter changes or Leigh syndrome-like pattern affecting brainstem and basal ganglia, similar to other genetic energy metabolic disorders [3, 11, 18]. Both two patients we reported in this study had developmental retardation, dystonia, feeding difficulties and malnutrition. Patient 1 also had nystagmus, elevated blood lactate and pyruvate. Serum alpha-hydroxybutyrate dehydrogenase and lactate dehydrogenase and creatine kinase isoenzymes in patient 2 were elevated. Both two patients had abnormal signals of bilateral globus pallidus on brian MRI. Patient 1 showed inverted lactate peaks in bilateral lesions on brian MRS, which was consistent with the results reported in the literatures.

The pathophysiology of this disease is still unclear. It has been hypothesized to be secondary to toxic accumulation of intermediate metabolites that exert brain toxicity and derange mitochondrial energy production. Previous studies have shown that ECHS1 mutation can induce accumulation of active metabolites of methacrylyl-CoA and acryloyl-CoA [7, 13]. SCEH protein has high specificity and activity to methacrylyl-CoA, which is a toxic compound that tends to form adducts with cysteine and causes cell damage. Therefore, the loss of SCEH enzyme activity may lead to the accumulation of this toxic compound and pathogenicity [9].

Evidence is emerging to support that,levels may correlate with disease severity. In retrospective analysis of mitochondrial encephalopathy and transient 3-methylglutaconic aciduria caused by ECHS1 deficiency, Huffnagel et al. suggested that S-(2-carboxypropyl) cysteine, S-(2-carboxypropyl) cysteine and N-acetyl-S-(2-carboxypropyl) cysteine might been useful diagnostic makers of SCEH deficiency [12]. For patients with mild forms of deficiency, only N-acetyl-S-(2-carboxypropyl) cysteine level is increased in the urine, suggesting that the metabolites of methacrylyl-CoA are important diagnostic markers of the mild and severe forms of ECHS1 deficiency. N-acetyl-S-(2-carboxypropyl)cysteine and its derivatives are formed from methacrylyl-CoA, so methacrylyl-CoA is also an important diagnostic marker for mild SCEHdeficiency [6, 12, 20, 21]. Peter et al. found that the content of 2,3-dihydroxy-2-methylbutyrate in urine of two SCEHdeficiency patients increased in the study of ECHS1mutations in LS suggesting that the metabolite may be a common biochemical change in SCEHdeficiency [1].

Fitzsimons et al. studied the role of SCEH in the metabolism of fatty acids and branched chain amino acids in four patients with ECHS1 gene mutation. The results of enzyme activity measurement and western blot analysis strongly indicated that there was a correlation between residual SCEH activity and severity of clinical symptoms [12, 16]. In this study, two patients received urine organic analysis, but no abnormality was found. The second urine metabolic test used urease method, and found 2, 3-dihydroxy-2-methylbutyric acid increased, which was related to the sensitivity of the detection method. 2,3-dihydroxy-2-methylbutyric acid in urine was only slightly elevated in patient 2 due to treatment. After further special urine examination was performed, S-(2-carboxypropyl) cysteamine was found increased significantly, which supported the diagnosis of SCEH deficiency and was consistent with gene diagnosis. The organic acid extraction technique in urine metabolism examination has low sensitivity in such patients and urease method can be used to improve the positive rate.

Three rare variants were detected by clinical exome sequencing of the probands.. Patient 1 had a compound heterozygous mutation of pathogenic variant c.414 + 1G > A and likely pathogenic variant c.161G > A, which was inherited from her father and mother respectively. Patient 2 has a homozygous mutation of c.74G > A. His mother carried a heterozygous mutation and his father had a gene deletion. c.161G > A ECHS1 variant has been previously reported by Haack et al. for the first time in the study of mitochondrial encephalopathy with cardiac involvement caused by ECHS1 deficiency. In his study, three patients in 10 families were found to carry c.161G > A mutation gene. All these three patients and patient 1 in our study had onset in infancy, accompanied by developmental retardation. Some patients had the same manifestations as patient 1, such as dystonia, nystagmus. Elevated serum lactate level, bilateral basal ganglia signal changes on brian MRI, and lactate peak on MRS were observed. Two of the three reported cases showed elevated urine S-(2-caboxypropyl)cysteamine concentration by urine specific metabolic screening. The c.161G > A variant has a population frequency of 0.000032 in East Asian. The pathogenic c.74G > A mutation is novel and never been reported before.

SCEH deficiency is a high mortality rate and poor prognosis disease. Most patients died of severe metabolic acidosis in infancy. So far, no standard therapy for ECHS1 deficiency exists for the treatment of SCEH deficiency. Some patients have received “cocktail therapy”, and the production of the toxic methacrylyl-CoA and its derivatives might be avoided with specific dietary regimens that reduce the workload on the valine catabolic pathway [9]. Some researchers have observed the increase of N-acetyl-S-(2-carboxypropyl) cysteine levels in patients with SCEH and HIBCH, suggesting that glutathione metabolism may be the cause of sulfhydryl-containing metabolites in urine. The combination of glutathione and glutathione S-transferase is an effective detoxification method for excretion of this abnormal metabolite through urine. Therefore, it is believed that supplementation of N-acetylcysteine may be beneficial to increase the content of glutathione, thereby eliminating toxic metabolites [18]. Some experts suggest that the combined approach of N-acetylcysteine supplementation and a restricted diet can be safely utilized in SCEH deficiency patients. In addition, there are literatures on ketogenic diet for SCEH deficiency [11, 16, 17], but the efficacy and safety of it remains yet to be studied. In this study, two patients were treated with cocktail therapy, low valine diet and rehabilitation therapy. Both of their motor functions were slightly improved after treatment.

In conclusion, SCEH deficiency caused by mutations of ECHS1 gene is a rare mitochondrial disease and patients typically present with psychomotor retardation, dystonia, nystagmus, cardiomyopathy, sensorineural hearing impairment, hyperlacticemia and Leigh syndrome-like cranial MRI in infants and young children. Elevated levels of 2,3-dihydroxy-2-methylbutyrate, S-(2-carboxypropyl) cysteine, S-(2-carboxypropyl) cysteine and N-acetyl-S-(2-carboxypropyl) cysteine can be diagnostic clues towards the disease spectrum of ECHS1 mutations in patients with an early-onset and mitochondrial encephalopathy. Early cocktail therapy, valine restriction diet and N-acetylcysteine supplementation can improve the prognosis of patients.

## Supplementary information

**Additional file 1:** CARE checklist.

## Data Availability

All data generated or analysed during this study are included in this published article [and its Additional file 1].

## Abbreviations

DCM: Dilated cardiomyopathy
HCM: Hypertrophic cardiomyopathy
N-acetyl-SCPC: N-acetyl-S-(2-carboxypropyl)-cysteine (N-acetyl-methacryl-l-cysteine
ND: Not determined
NL: Not listed
SCPC: S-(2-carboxypropyl)cysteine
SCPCM: S-(2-caboxypropyl)cysteamine
