# Supplementary material for: Clinical, biochemical and metabolic characterization of patients with short-chain enoyl-CoA hydratase(ECHS1) deficiency: two case reports and the review of the literature
Source: BMC Pediatr. 2020 Feb 3;20:50. doi: 10.1186/s12887-020-1947-z (PMC6996175; doi:10.1186/s12887-020-1947-z)
Supplement: Supplementary file 1 — Additional file 1: CARE checklist. [file 12887_2020_1947_MOESM1_ESM.pdf]

CARE case report guidelines

CARE Checklist (2013) of information to include when writing a case report

(cc) BY-NC-ND

Topic	Item	Checklist item description	Reported on Page
**Title**	1	The words "case report" should be in the title along with the area of focus	page 3
**Key Words**	2	2 to 5 key words that identify areas covered in this case report.	page 3
**Abstract**	3a	Introduction—What is unique about this case? What does it add to the medical literature?	abstract, page 3
	3b	The main symptoms of the patient and the important clinical findings	abstract, page 3
	3c	The main diagnoses, therapeutics interventions, and outcomes	abstract, page 4
	3d	Conclusion—What are the main "take-away" lessons from this case?	abstract, page 4
**Introduction**	4	One or two paragraphs summarizing why this case is unique with references	background, page 4
**Patient Information**	5a	De-identified demographic information and other patient specific information	background, page 4
	5b	Main concerns and symptoms of the patient	case presentation, page 5, 6
	5c	Medical, family, and psychosocial history including relevant genetic information (also see timeline).	case presentation, page 5, 6
	5d	Relevant past interventions and their outcomes	case presentation, page 11
**Clinical Findings**	6	Describe the relevant physical examination (PE) and other significant clinical findings.	case presentation, page 5, 6
**Timeline**	7	Important information from the patient's history organized as a timeline	case presentation, page 5, 6
**Diagnostic Assessment**	8a	Diagnostic methods (such as PE, laboratory testing, imaging, surveys).	case presentation, page 5, 6,7
	8b	Diagnostic challenges (such as access, financial, or cultural)	N/A
	8c	Diagnostic reasoning including other diagnoses considered	case presentation, page 5, 6,7
	8d	Prognostic characteristics (such as staging in oncology) where applicable	discussion page 12
**Therapeutic Intervention**	9a	Types of intervention (such as pharmacologic, surgical, preventive, self-care)	discussion page 11
	9b	Administration of intervention (such as dosage, strength, duration)	N/A
	9c	Changes in intervention (with rationale)	discussion page 11
**Follow-up and Outcomes**	10a	Clinician and patient-assessed outcomes (when appropriate)	discussion page 12
	10b	Important follow-up diagnostic and other test results	discussion page 12
	10c	Intervention adherence and tolerability (How was this assessed?)	N/A
	10d	Adverse and unanticipated events	N/A
**Discussion**	11a	Discussion of the strengths and limitations in your approach to this case	N/A
	11b	Discussion of the relevant medical literature.	discussion page11
	11c	The rationale for conclusions (including assessment of possible causes)	discussion page 12
	11d	The primary "take-away" lessons of this case report	discussion page 12
**Patient Perspective**	12	When appropriate the patient should share their perspective on the treatments they received	discussion page 12
**Informed Consent**	13	Did the patient give informed consent? Please provide if requested	Yes ☑ No ☐
